# Screening the Best Compatibility of* Selaginella moellendorffii* Prescription on Hyperuricemia and Gouty Arthritis and Its Mechanism

**DOI:** 10.1155/2019/7263034

**Published:** 2019-07-11

**Authors:** Xue-yan Zhang, Jing Cheng, Ping Zhao, Ke-li Chen, Juan Li

**Affiliations:** Key Laboratory of Ministry of Education on Traditional Chinese Medicine Resource and Compound Prescription and Hubei Key Laboratory of Resources and Chemistry of Chinese Medicine, Hubei University of Chinese Medicine, Wuhan, Hubei 430065, China

## Abstract

**Objectives:**

The* Selaginella moellendorffii *prescription (SMP) consists of S. moellendorffii Herba (SM), Smilacis glabrae Rhizoma (SGR), and Plantaginis Semen (PS). It has been commonly used to treat hyperuricemia and acute gouty arthritis as a hospital preparation. This study was aimed at investigating the best compatibility ratio of SMP on hyperuricemia and gouty arthritis and getting better insight of the possible mechanism.* Methods. In vitro*, anti-inflammatory activity of SMP was evaluated by lipopolysaccharide (LPS) induced RAW264.7 cells. The release of nitric oxide (NO) was screened by Griess assay, and NF-*κ*B p65 and NLRP3 proteins expression was examined by immunofluorescence assay. Then, the levels of creatinine (Cr), blood urea nitrogen (BUN), and uric acid (UA) were detected in mice induced by potassium oxonate, and the paw oedema, inflammatory mediators, and histological examination were analyzed in rats induced by monosodium urate (MSU). HPLC method was employed to investigate the chemical profile of this preparation.* Results. In vitro, *SMP-3 (the ratio of SMP:SGR:PS was 3:1:1) exhibited the most potent anti-NO production activity without obvious toxicity. This anti-inflammatory effect was associated with suppression of NF-*κ*B p65 nuclear translocation and NLRP3 protein expression. In animal experiments, the levels of BUN and Cr in SMP-3 group were lower than other extract groups, and the level of UA was also remarkably decreased by SMP-3 in hyperuricemic mice (*P*<0.01). Besides, SMP-3 extract was able to prevent the paw edema, reduce gouty joint inflammatory features, and decrease the levels IL-1*β*, PGE-2, IL-8, and NO in gouty arthritis rats. Furthermore, 6-C-*β*-D-xylopyranosyl-8-C-*β*-D-glucopyranosyl, apigenin, and astilbin were identified from SMP-3 extract.

**Conclusions:**

In summary, SMP-3 may be a potential therapeutic agent for the prevention of hyperuricemic and gout.

## 1. Introduction

Gout is the chronic inflammatory arthritis which is caused by hyperuricemia and the deposition of MSU crystals in the articular cavity [1]. According to the clinical manifestation of gout, it can be divided into four stages: asymptomatic period, high blood UA level stage without symptoms, acute stage, and chronic stage [2]. Generally, hyperuricemia is defined as UA level > 7.0 mg/dL, which derives from the increase in purine metabolism and impairment of renal excretion of UA [3, 4]. Meanwhile, hyperuricemia is the most dominant factor in gout occurrence and clearly associated with a variety of comorbidities, including cardiovascular diseases [5], chronic kidney disease [6], and urolithiasis and metabolic syndrome [3]. Thus, the major purpose of clinical treatment of gout is to reduce the UA level and inflammatory response [7].

Currently, the drugs used for gout treatment include allopurinol (ALL), probenecid, colchicine (COL), interleukin-1 (IL-1) inhibitors, glucocorticoids, and other anti-inflammatory drugs [8, 9]. Although these drugs are generally effective, they will increase the risk of ADR in some patients who have preexisting renal, liver, gastrointestinal, and cardiovascular diseases [10, 11].

SMP, a traditional herbal formula that consists of SM, SGR and PS, has been used as a hospital preparation in Huanggang Hospital of Traditional Chinese Medicine, which confirmed effectiveness for the treatment of gout. Besides, SM has been used for treatment of bleeding and chronic inflammation, such as arthritis, gonorrhea, and hepatitis [12, 13]. Our previous studies showed SM extract possessed activities of antihyperuricemia, anti-inflammation, and xanthine oxidase (XOD) inhibition [14]. SGR is described to be effective in strengthening bones and muscles, getting rid of rheumatism and easing joint movement in* Compendium of Materia Medica*. It has been used in the clinical treatment of gout and hyperuricemia for thousands of years for its significant inhibitory effect on serum UA level. Its mechanism may be relevant to inhibit the uric acid transporter 1 (URAT1) gene expression [15]. Additionally, PS, another major component of the SMP formula, was able to suppress the activity of cyclooxygenase (COX-2), catalyzing prostaglandin synthesis [16]. Aucubin, its main constituent, showed an inhibitory effect on 12-O-tetradecanoylphorbol acetate (TPA)-induced mouse ear oedema [17]. Based on the traditional uses and known functionalities of their phytochemical constituents, this study was designed to investigate the best compatibility ratio of SMP on hyperuricemia and gouty arthritis and get an insight of the possible mechanism.

## 2. Materials and Methods

### 2.1. Chemicals and Reagents

Lipopolysaccharide (LPS; 0111:B4; L4391), allopurinol (A8003), colchicine (C3915), potassium oxonate (156124), monosodium urate (MSU; U2875), 3-(4,5-dimethylthiazol-2-yl)-2, 5-diphenyltetrazolium bromide (MTT; M5655), and dimethyl sulfoxide (DMSO; D2650) were purchased from Sigma Aldrich (USA). The UA (012-2-1), NO (A012-1-2), IL-1*β* (H002), IL-6 (H006), IL-8 (H008), and PGE-2 (H099) ELISA kits were purchased from NanJing Jiancheng Bioengineering Institute Co., Ltd. (Nanjing, China). Dulbecco's modified Eagle's medium (DMEM; SH 30022.01) and penicillin/streptomycin (15140-122) were obtained from Gibco (USA). Fetal bovine serum (FBS; SV 30087.02) was purchased from HyClone (USA). Polyclonal rabbit antibodies against rats p65(GB11142) and FITC-conjugated goat anti-rabbit IgG (GB22303) were purchased from Servicebio (Wuhan, China). Polyclonal rabbit antibodies against rats NLRP3 (A5652) was purchased from (Wuhan, China).

### 2.2. Plant Materials and Extract Preparation

All raw materials were purchased from Jointown Pharmaceutical Group Co., Ltd., China. All the herbal medicines in the preparation were authenticated by authors (*Prof.* Ke-li Chen). Specimens of these materials were deposited in the herbarium, Hubei University of Chinese Medicine, China.

First, all the herbal medicines were pulverized and screened through 24-size mesh. Then, 50 g of materials were immersed in 500 mL distilled water. The solution was heated to 100°C and lasted for 1 h three times. The combined aqueous extract was filtered and lyophilized into powder. The different mass ratio of SMP was shown in the Table 1. The extraction rates were about 20%.

### 2.3. Cell Culture and Treatments

#### 2.3.1. Cell Culture and Stimulation

The mouse monocyte/macrophage cell line RAW264.7 was obtained from China Center for Type Culture Collection of Wuhan University (Wuhan, China). RAW264.7 cells were routinely cultured for 24 h in DMEM supplemented with 10% FBS and 1% streptomycin/penicillin at 37°C in a humidified incubator with 5% CO_2_ under saturating humidity. Subsequently, the cells were preincubated with or without SMP for 2 h before LPS (0.5 *μ*g/ml) 24 h treatment.

#### 2.3.2. Cell Viability Assay

RAW264.7 cells were seeded in a 96-well plate at a density of 1×10^5^ /ml and a volume of 100 *μ*l/well. After incubation for 24 h at 37°C, the cells were treated with or without SMP (400 *μ*g/mL) and then treated with LPS for an additional 24 h. This was followed by the addition of 5 mg/ml MTT solution to each well; the plates were further incubated for 4 h at 37°C. The supernatant was removed, and 200 *μ*l DMSO was added to each well to solubilize the water-insoluble purple formazan crystals. The absorbance at a wavelength of 570 nm was measured using a microplate reader (BioTek Instruments, Inc., USA).

#### 2.3.3. NO Determination

NO secretion was determined using the Griess assay. Briefly, the RAW264.7 cells were treated with SMP for 2 h before LPS treatment. The culture supernatant was collected and mixed with an equal amount of Griess reagent in a 96-well plate. The absorbance at 540 nm was determined using a microplate reader.

#### 2.3.4. Immunofluorescence Assay

RAW264.7 cells were cultured on sterile coverslips in 6-well plates at a density of 4×10^5^/ml and supplemented and stimulated with LPS or SMP as described above. The cells were fixed with 4% paraformaldehyde for 10 min at room temperature and rinsed in PBS two times and permeabilized in 0.1 % Triton-X100 in PBS for 10 min. After washing several times, the cells were blocked in PBS containing 6% goat serum. Subsequently, the cells were incubated with the primary antibody (diluted 1:200) at 4°C overnight. After the cells were washed with PBS, FITC-conjugated anti-rabbit IgG (diluted 1:200) was applied for 1 h. This process was performed in the dark at room temperature, and fluorescence was visualized using a fluorescence microscope (IX73; Olympus, Tokyo, Japan) at a magnification of ×400.

### 2.4. Animals and Treatments

#### 2.4.1. Animals

SPF Kunming male mice (18-22 g) and male Sprague-Dawley rats (180-220 g) were bought from the Hubei Provincial Center for Disease Control and Prevention, Wuhan, China. All animals were housed under standard laboratory conditions, including the temperature 24±2°C and humidity 55±15% with a normal light-dark cycle. All animals were allowed to acclimatize to the environment for 1 week before experiment. The animal experimental protocol was approved by the Institutional Animal Care and Use Committee and the local experimental Ethics Committee (Laboratory Animal Certificate no. SYXK 2012-0068)

#### 2.4.2. Animal Model of Hyperuricemia in Mice

*In vivo* potassium oxonate (a uricase inhibitor) induced hyperuricemic animal model was adopted, with some modification [18]. The experimental mice were randomly divided into nine groups (n=8). Control animals were kept without additional treatment. The hyperuricemic control groups of mice were given intraperitoneally with potassium oxonate (250 mg/kg) and adenine (300 mg/kg). The treatment groups received the same dose of potassium oxonate and adenine 1 h before intragastric administration of SMP extracts (102 g/kg/day raw materials) or allopurinol (20 mg/kg), respectively. The samples were administrated to corresponding groups by oral gavage once a day for 2 weeks. Then, mice were anesthetized and then blood was collected from retro-orbital sinus puncture. The blood samples were allowed to clot for approximately 1 h at room temperature and then centrifuged to obtain the serum (supernatant). All samples were frozen at -80°C.

#### 2.4.3. Animal Model of Acute Gouty Arthritis in Rats

In order to evaluate the preventive efficacy of SMP-3 on gout, the experimental model was executed as mentioned previously [19]. The rats were equally divided into 4 groups (n=8). In group 1, the normal control was given orally only normal saline for 1 week. Group 2 served as gout animal model control. Groups 3 and 4 were treated with colchicine (0.3 mg/kg) and SMP-3 (52 g/kg/day raw materials) orally for 1 week, respectively. Except normal control group, each group injected with 100 *μ*l suspension of MSU (100 mg/ml) into the right hind paw by intra-articular injection 1 h before the corresponding samples treatments orally. The inflammation was quantified by measuring the ankle circumference with a tape at 0 h, 2 h, 3 h, and 6 h after MSU crystal injections. At the end of experimental period, all rats were anesthetized with 0.3% nembutal (10 ml/kg), in order to allow blood collection from abdominal aorta. Serum was separated and stored at -20°C until assay for serum biochemical assays of NO, IL-6, IL-8, IL-1*β*, and PGE-2 quantification in accordance with manufacturer's protocol.

#### 2.4.4. Histopathological Examination of Ankle Joints

The right ankle joint of rats was cut, stripped off the skin, washed in saline, sucked into the surface, and placed in 10% buffered neutral formalin. After that, ankle joints were dehydrated, embedded in paraffin, and then cut into 5 mm thick paraffin sections. Subsequently, paraffin sections were stained with hematoxylin and eosin (HE) using standard techniques.

### 2.5. HPLC Analysis

HPLC analysis of extract was performed on a Dionex HPLC system with P680 pump, a Welchrom-C18 (4.6 mm×250 mm, 5 *μ*m), and a UVD 170 U variable wavelength UV-Vis detector. Data were collected and processed using “Chromeleon version 6.0” software. The mobile phase consisted of methanol (A) and water including 2% tetrahydrofuran and 0.1% trifluoroacetic acid (B). The gradient program was as follows: 18-25% A in 0-5 min, 25-50% A in 5-55 min, 50-100% A in 55-58 min, 100% A in 58-60 min, and 100-18% A in 60-65 min. The flow rate was 1.0 ml/min, and column temperature was maintained at 30°C. The injection volume was 20 *μ*l. The detector was set at 330 nm for acquiring chromatograms.

### 2.6. Statistical Analysis

The results were expressed as the mean ± standard error of the mean (SEM). Differences between the mean values for the individual groups were assessed by one-way analysis of variance (ANOVA), with Newman-Keuls Multiple Comparison Test.* P*<0.05 was considered to indicate a statistically significant difference.

## 3. Results

### 3.1. Effects of SMP on LPS-Induced NO Production in RAW264.7

**Figure 1:**
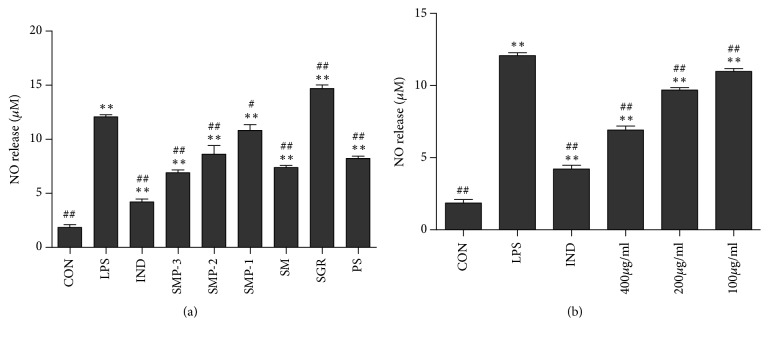
(a) Effects of SMP on NO production in RAW264.7 cells. (b) Effect of SMP-3 on NO production in RAW264.7 cells. Values shown are mean ± SEM from six independent experiments. ^*∗∗*^*P* < 0.01 and ^*∗*^*P* < 0.05 compared with the normal control group; ^#^*P* < 0.05 and ^##^*P* < 0.01 compared with the LPS control group. CON, normal control; LPS, LPS-stimulated cells; IND, LPS-induced cells treated with indomethacin. SMP-3, SMP-2, SMP-1, SM, SGR, and PS were expressed as LPS-induced cells treated with SMP-3, SMP-2, SMP-1, SM, SGR, and PS, respectively.

The MTT results showed that all the extracts were nontoxic to RAW 264.7 cells at 400 *μ*g/ml, except PS which possessed a weak cytotoxicity. As shown in Figure 1(a), when the cells were exposed to LPS for 24 h, the NO production in the cell supernatant was significantly increased (*P*<0.01). All extracts potently inhibited NO production except for SGR. Meanwhile, SMP-3 exhibited the most promising anti-NO production activity. As shown in Figure 1(b), the NO production after SMP-3 treatment was decreased significantly in a dose dependent manner.

### 3.2. Effects of SMP-3 on LPS-Stimulated NF-*κ*B p65 and NLRP3 Protein Expression in RAW264.7

**Figure 2:**
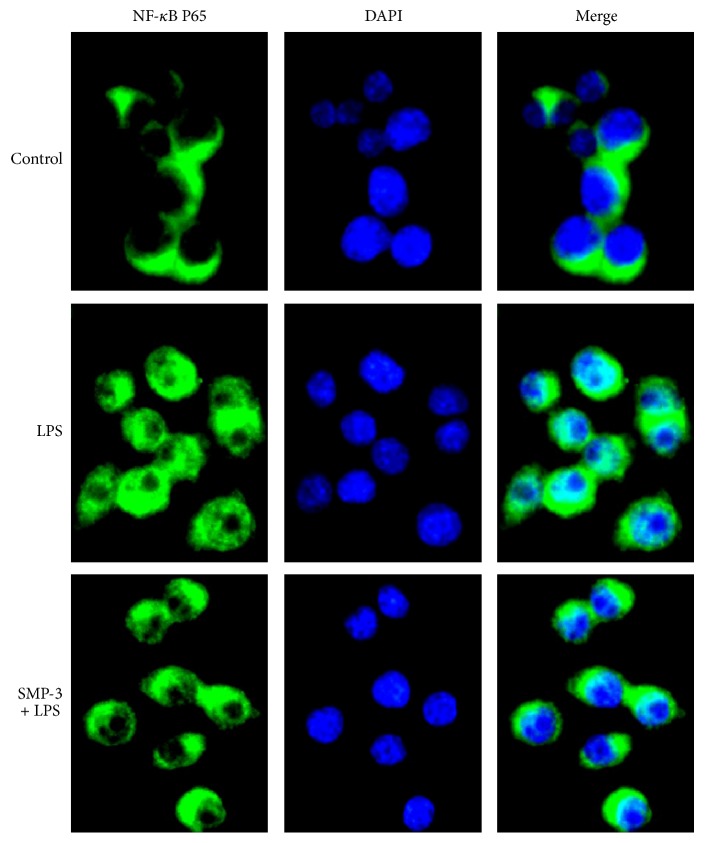
Effect of SMP-3 on NF-*κ*B p65 protein expression in LPS-induced RAW264.7 cells. Cells were visualized using a fluorescence microscope (×400). Control, normal control cells; LPS, LPS-stimulated cells; SMP-3 +LPS, LPS-induced cells treated with SMP-3.

**Figure 3:**
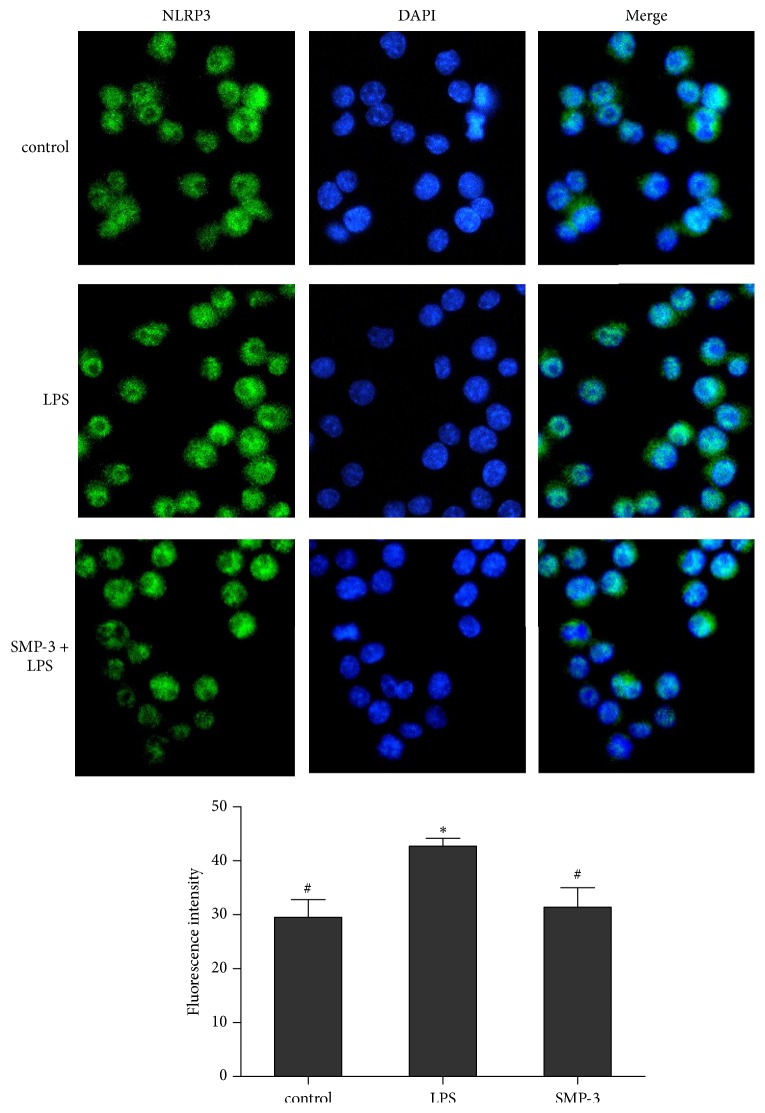
Effect of SMP-3 on NLRP3 protein in LPS-induced RAW264.7 cells. Cells were visualized using a fluorescence microscope (×400). Control, normal control cells; LPS, LPS-stimulated cells; SMP-3 +LPS, LPS-induced cells treated with SMP-3.

Transcription factor NF-*κ*B p65 is known to be pivotal mediator of proinflammatory cytokine and inflammatory enzyme expressions in the inflammatory disorders like arthritis [20]. As shown in Figure 2, LPS stimulation induced the nuclear translocation of p65 in RAW264.7 cells, but the pretreatment of SMP-3 significantly attenuated NF-*κ*B p65 activation. The NLRP3 inflammasome is a key inducer of inflammation in response to pathogens and innate immune stimuli [21]. The results showed that LPS increased the protein expression of NLRP3, but SMP-3 pretreatment inhibited the level of NLRP3 protein induced by LPS (Figure 3).

### 3.3. Effects of SMP on Serum Levels of BUN and Cr

**Figure 4:**
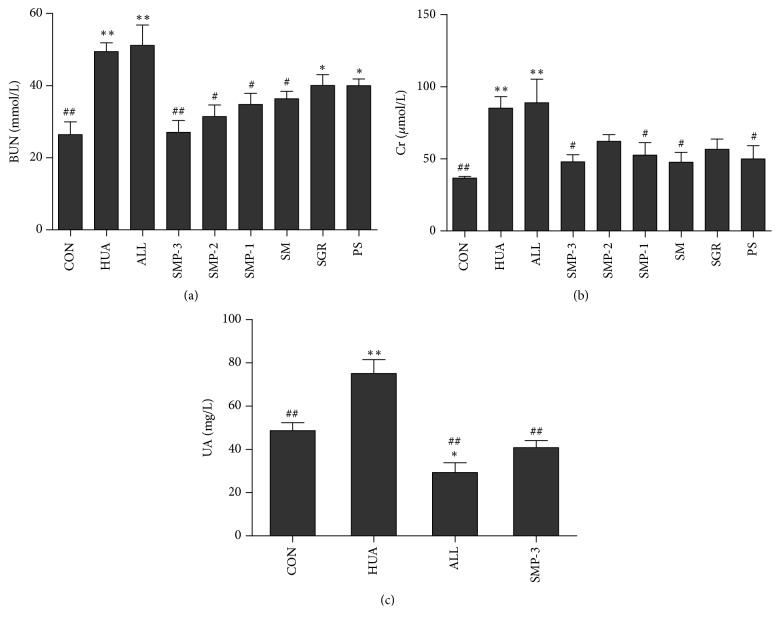
The effects of SMP on BUN (a) and Cr (b) in the serum of hyperuricemic mice. (c) The effect of SMP-3 extract on UA in the serum of hyperuricemic mice. Values shown are mean ± SEM (*n*=8). ^*∗∗*^*P* < 0.01 and ^*∗*^*P* < 0.05 compared with the normal control group; ^#^*P* < 0.05 and ^##^*P* < 0.01 compared with the hyperuricemic control group. CON, normal control group; HUA, hyperuricemic control group; ALL, allopurinol group. SMP-3, SMP-2, SMP-1, SM, SGR, and PS were expressed as SMP-3, SMP-2, SMP-1, SM, SGR, and PS group, respectively.

As shown in Figures 4(a) and 4(b), the serum level of BUN and Cr in hyperuricemic control group was increased significantly comparing with the control group (*P*<0.01). Moreover, after SMP treatment, the serum level of BUN and Cr was decreased as compared with the hyperuricemic control group, especially the SMP-3 group (*P*<0.01). However, there was no significant difference in BUN and Cr between HUA group and ALL group (*P*>0.05).

### 3.4. Antihyperuricemic Effect of SMP-3 on Hyperuricemic Mice

As shown in Figure 4(c), the hyperuricemic control group exhibited significantly higher UA level comparing with control group (*P*<0.01). As compared with hyperuricemic group, SMP-3 administration significantly decreased UA level (*P*<0.01), which was almost the same as the control group. ALL as a positive control reduced the level of UA in hyperuricemic mice (*P*<0.01), but even lower than normal control group.

### 3.5. Effect of SMP-3 on MSU-Induced Paw Edema in Rats

**Figure 5:**
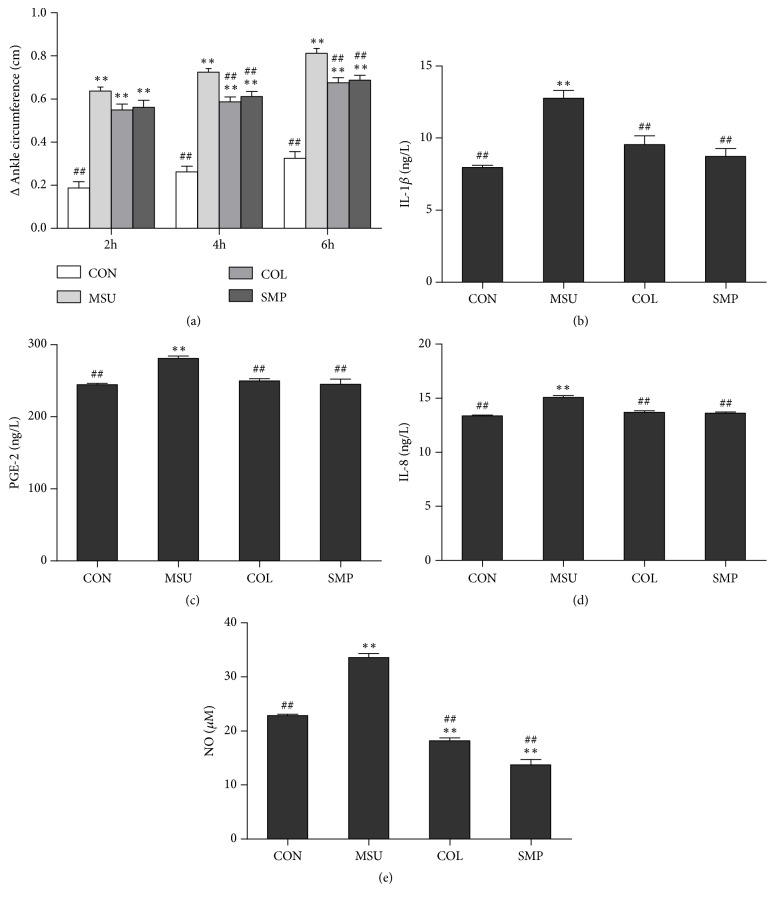
The effects of SMP-3 extract on paw edema (a), IL-1*β* (b), PGE-2 (c), IL-8 (d), and NO (e) level in gouty arthritis rats. Values shown are mean ± SEM (*n*=8). ^*∗∗*^*P* < 0.01 and ^*∗*^*P* < 0.05 compared with the normal control group; ^#^*P* < 0.05 and ^##^*P* < 0.01 compared with the MSU control group. CON, normal control group; MSU, monosodium urate control group; COL, colchicine group; SMP-3, SMP-3 group.

To evaluate the effect of SMP-3 on MSU-induced paw edema, we measured the circumference of ankle joint on rats. As shown in Figure 5(a), the ankle circumference of rats in MSU control group was found to be significantly increased compared to the normal group (*P*<0.01). In contrast, SMP-3 and COL pretreatment significantly suppressed the MSU-induced paw edema formation (*P*<0.01).

### 3.6. Effects of SMP-3 on MSU-Induced Inflammatory Cytokine Production in Rats

To investigate the anti-inflammatory effects of SMP-3, the production of IL-1*β*, PGE-2, IL-8, and NO in serum was examined. As shown in Figures 5(b)–5(e), the level of IL-1*β*, PEG-2, IL-8, and NO induced by MSU exhibited remarkable increase compared to that induced by normal saline (*P* < 0.01), while the increase in IL-1*β*, PEG-2, IL-8, and NO level was prevented by both COL and SMP-3 (*P* < 0.01).

### 3.7. Effect of SMP-3 on Histopathological Change in Ankle Joint

**Figure 6:**
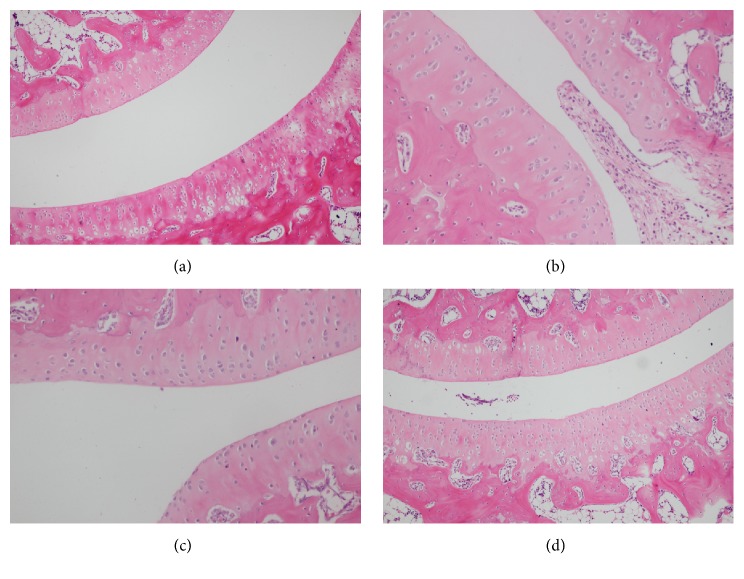
Histopathological examination of ankle joint. (a) Normal control group; (b) MSU control group; (c) colchicine group; (d) SMP-3 group.

As shown in Figure 6, the articular cavity of the normal control group was observed in Figure 6(a); the result indicated that the intra-articular injection of normal saline could result in only a slight inflammation. In model group, a large number of neutrophil infiltration and shed synovial membrane could be seen in articular cavity, showing that UA sodium can cause leukocyte invasion to induce inflammation and synovial tissue injury in Figure 6(b). However, after the acute gouty arthritis rats treated with COL and SMP-3 treatment could reduce the invasion of the leukocyte in articular cavity of acute gouty arthritis rats (Figures 6(c) and 6(d)).

### 3.8. HPLC Analysis

**Figure 7:**
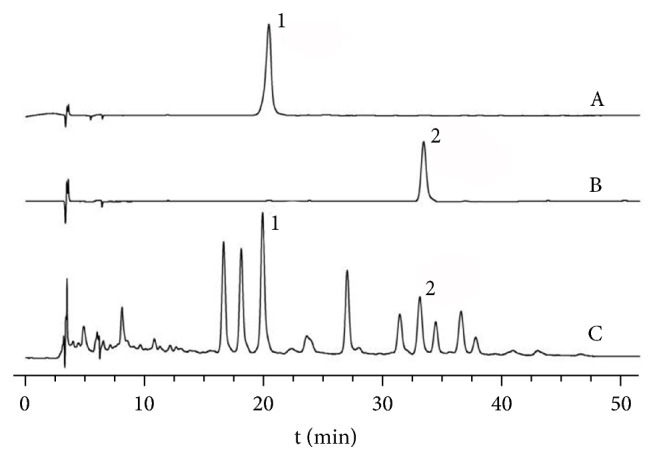
HPLC chromatogram of SMP-3 extract. The peaks indicate the following: 1: 6-C-*β*-D-glucopyranosyl-8-C-*β*-D-xylopyranosylapigenin; 2: astilbin.

HPLC chromatogram was applied for examining constituents from SMP-3 aqueous extract; HPLC chromatograms revealed the two marker components in SMP-3. These components were identified as 6-C-*β*-D-glucopyranosyl-8-C-*β*-D-xylopyranosylapigenin and astilbin (Figure 7).

## 4. Discussion

Gout is a crystal-deposition related disease, and epidemiological evidence suggested its incidence and prevalence increased substantially in recent years [22]. Traditional Chinese medicine has gained growing interest in the prevention and treatment of gout. According to the theory of Chinese medicine, excessive internal dampness and phlegm turbidity are the key cause of hyperuricemia [23]. PS and RSG in this preparation or diet will accelerate the excretion of dampness as well as phlegm turbidity, and alleviating xeransis, detoxification, respectively. The combination of them will do help to the recovery of joint movement functions [14]. Furthermore, our previous research has confirmed the efficiency of SM treatment by lowering UA level and reducing the inflammatory reaction in the gout rats model [13]. In this study, we found that SMP-3, which contained higher ratio of SM, had a significant inhibitory effect of NO production. These results indicated that SM was “monarch drug” in the prescription and played the most important role in the formula, while PS and RSG increased the effectiveness of the monarch drug.

We further investigated the anti-inflammatory mechanism of the preparation by employing LPS-induced RAW 264.7. The results showed that the anti-inflammatory effect of this preparation was closely related to downregulation of several inflammation-related proteins to elucidate the possible mechanism. NF-*κ*B, the key mediator of immunity, plays a critical role for the priming of the NLRP3 inflammasome. Liberated NF-*κ*B such as p50/p65 dimer translocates into the nucleus to regulate the transcription of proinflammatory mediators [24]. In this study, the results suggested that the expression of p65 and NLRP3 proteins was markedly suppressed after treatment with SMP-3. These indicate that SMP-3 might suppress the translocation of NF-*κ*B p65 to suppress NLRP3 activation.

During the progression of gouty arthritis, MSU crystals stimulate monocytes and macrophages to release several inflammatory mediators [25]. Therefore, anti-inflammatory treatment which showed promising effect on inhibiting the production of proinflammatory cytokines (IL-1*β* and IL-8) and inflammatory mediators (PGE-2 and NO) was demonstrated to benefit the patients by alleviating the syndromes, such as swelling, pain, and inflammations. Meanwhile, the real cause of this disease, MSU crystal deposition in and around the joints, which caused by longstanding hyperuricemia [2] was still not resolved. Controlling UA level is of the great importance for dissolving pathogenic crystal deposits. In the present study, the results showed that SMP-3 could significantly reduce UA level, prevent the paw edema, reduce gouty joint inflammatory features, and decrease the release of IL-1*β*, PGE-2, IL-8, and NO. These indicate that SMP-3 had good effects on hyperuricemia and acute gouty arthritis by decreasing UA and inflammatory cytokines levels. These results illustrated that the anti-inflammatory activity of SMP-3 may contribute to antihyperuricemia and anti-gout.

Previous phytochemical studies about these 3 herbs focused on bioflavonoids, such as chrysoeriol [13], amentoflavone [26], and ginkgetin [27] isolated from SM, astilbin isolated from RSG [28], and aucubin isolated from PS [17]. In this study, 6-C-*β*-D-glucopyranosyl-8-C-*β*-D-xylopyranosylapigenin and astilbin were identified from SMP extract. Astilbin (taxifolin-3-O-rhamnoside) has been reported activities of hepatoprotection [29], anti-inflammation [30], and antioxidation [31], while 6-C-*β*-D-glucopyranosyl-8-C-*β*-D-xylopyranosylapigenin was isolated from SM in our previous works. However, further investigations are warranted in order to identify the active components of the aqueous extract from SMP-3, responsible for the observed antihyperuricemia and anti-gout effects.

In summary, SMP-3 extract had powerful anti-inflammation activity* in vitro*, by suppressing of NO production, p65 nuclear translocation, and NLRP3 expression.* In vivo*, SMP-3 was able to prevent the paw edema, reduce gouty joint inflammatory features, and decrease the release of IL-1*β*, PGE-2, IL-8, and NO levels in gouty arthritis rats. In addition, SMP-3 will contribute to the prevention and slow down the progression of gout partially through reducing serum UA level in mice induced by potassium oxonate. The results of this study will pave the way for better treatment and prevention of gout.

## Figures and Tables

**Table 1:** The different mass ratio of SMP.

Formulas	SM	SGR	PS
SMP-1	1	1	1
SMP-2	2	1	1
SMP-3	3	1	1
SM	1	0	0
SGR	0	1	0
PS	0	0	1

## Data Availability

The datasets generated during and/or analyzed during the current study are available from the corresponding author on reasonable request.

## Abbreviations

SMP: Selaginella moellendorffii prescription
SM: Selaginella moellendorffii Herba
SGR: Smilacis glabrae Rhizoma
PS: Plantaginis Semen
LPS: Lipopolysaccharide
NO: Nitric oxide
Cr: Creatinine
BUN: Blood urea nitrogen
UA: Uric acid
MSU: Monosodium urate
ALL: Allopurinol
COL: Colchicine
IND: Indomethacin.

## Supplementary Materials

Supplementary Materials The effect of SMP on Cr level in the serum of hyperuricemic mice. It was combined into Figure 4 in this manuscript.
