# Supplementary figures and images for: Screening the Best Compatibility of Selaginella moellendorffii Prescription on Hyperuricemia and Gouty Arthritis and Its Mechanism
Source: Evid Based Complement Alternat Med. 2019 Jul 11;2019:7263034. doi: 10.1155/2019/7263034 (PMC6657646; doi:10.1155/2019/7263034)

Graphic Abstract

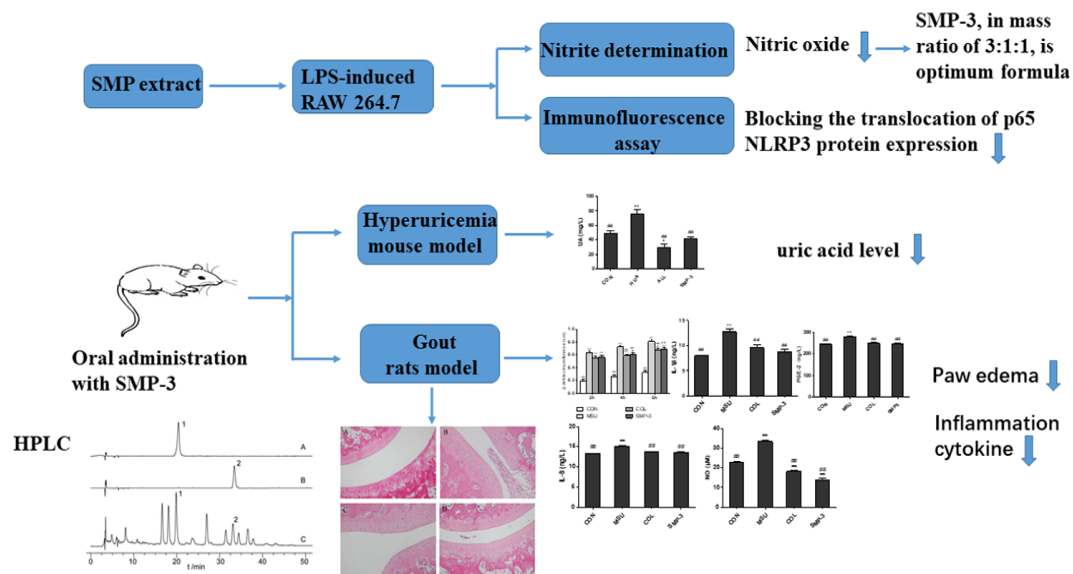

Supplement: Supplementary Materials — The effect of SMP on Cr level in the serum of hyperuricemic mice. It was combined into Figure 4 in this manuscript. [file 7263034.f1.pdf]
